# Intestinal microbiota could transfer host Gut characteristics from pigs to mice

**DOI:** 10.1186/s12866-016-0851-z

**Published:** 2016-10-11

**Authors:** H. Diao, H. L. Yan, Y. Xiao, B. Yu, J. Yu, J. He, P. Zheng, B.H. Zeng, H. Wei, X.B. Mao, D.W. Chen

**Affiliations:** 1 Institute of Animal Nutrition, Sichuan Agricultural University, Key Laboratory for Animal Disease-Resistance Nutrition of China Ministry of Education, Xinkang Road 46#, Ya’an, Sichuan Province 625014 People’s Republic of China; 2 Department of Laboratory Animal Science, College of Basic Medical Sciences Third Military Medical University, Gaotanyan Street, Chongqing, 400038 People’s Republic of China

**Keywords:** Pig breeds, Intestinal development, Digestive and absorptive activities, Germ-free mice, Fecal microbiota transplantation

## Abstract

**Background:**

The present study was conducted to compare the differences in gut microbiota composition and gut-phenotypes among pig breeds, and determine whether these differences would transmit to mice colonized with fecal microbiota of different pig breeds. A total of 24 1-day-old germ-free BALB/C mice were divided into 3 groups (TFM, YFM and RFM), which were transplanted with intact fecal microbiota of Tibetan pig (TP), Yorkshire pig (YP) and Rongchang pig (RP), respectively.

**Results:**

Results showed that different pig breeds exhibited distinct gut microbiota profile based on high-throughput pyrosequencing. YP exhibited a lower *Firmicutes/Bacteroidetes* ratio and apparent genera differences compared with RP and TP, and higher levels of bacteria from *Spirochaetes* were observed in TP compared with RP and YP (*P* < 0.05). Transplanted porcine microbiota into GF mice replicated the phenotypes of pig donors. Moreover, the three groups of donor pigs and their mice recipients exhibited different intestinal index and morphology. TP and RP had higher intestinal weight and relative CDX2 mRNA expression in ileum than YP, and longer intestine, higher villus height of duodenum and jejunum were observed in TP compared with YP and RP (*P* < 0.05). TP exhibited higher GLP-2 mRNA expression in duodenum and jejunum than RP (*P* < 0.05). Similarly, YFM had lower intestine weight and CDX2 mRNA expression in ileum than TFM and RFM (*P* < 0.05). The intestine length in TFM was longer than that in RFM, and TFM had higher villus height in duodenum and jejunum and GLP-2 mRNA expression in ileum than the other two groups (*P* < 0.05). Besides, the digestive and absorptive ability was different among the three groups in donor pigs and mice recipients. YP had higher jejunal lactase and maltase activities than TP and RP, while TP had higher activities of jejunal ATPase, γ-GT, and relative SGLT1 mRNA expression in duodenum and jejunum than YP and RP (*P* < 0.05). Likewise, YFM had higher jejunal sucrase and maltase activities than TFM and RFM, whereas higher jejunal γ-GT activity and relative SGLT1 mRNA expression in duodenum and ileum were observed in TFM compared with YFM and RFM (*P* < 0.05). In addition, Tibetan pigs-derived microbiota improved gut barrier in mice recipients. The concentration of MDA in YP was higher than that in TP and RP (*P* = 0.078), and the relative ZO-1 mRNA expression in ileum in TP was higher than that in YP (*P* < 0.05). Likely, compared with TFM and RFM, YFM exhibited increasing MDA concentration in jejunum (*P* = 0.098), and the relative ZO-1 mRNA expression in duodenum and ileum in TFM were higher than that in YFM (*P* < 0.05).

**Conclusions:**

There were huge differences in gut microbiota composition and gut characteristics among pig breeds, and gut microbiota could partially convey host gut characteristics from pigs to mice.

**Electronic supplementary material:**

The online version of this article (doi:10.1186/s12866-016-0851-z) contains supplementary material, which is available to authorized users.

## Background

Gut microbiota is a category of microorganisms inhabiting the mammalian gastrointestinal tract and participates in most host’s physiological processes from nutritional status to behavior and stress response [1]. Indeed, a broad range of biological functions that the host couldn’t otherwise accomplish are performed by this special organ, including providing nutrients, modulating gastrointestinal development and shaping immune system [2]. The commensal microbiota is highly variable from individual to individual, and the different regions of the host intestine harbor specific microbial communities altering in density and diversity [3]. Accumulating evidence indicates that diet, environment, host genotypes and phenotypes can strongly influence the composition of the gut microbiota in various mammals [4–7]. In the studies of monozygotic or dizygotic twin pairs, huge differences in the gut microbiota profiles were found between healthy twins and twins with malnourishment or obesity [8, 9], and these modifications can also be brought about by adding or deleting one gene to a model host organism [10, 11]. These researches strengthened the concept that distinct gut microbiota compositions are existed in host with different genotypes or phenotypes. The Tibetan pig (TP) is a typical native pig breed from Tibetan plateau, and is characterized by stronger adaptability and disease resistance [12]. The Rongchang pig (RP) is also a typical native pig breed from China, and is characterized by better meat quality [13]. While the Yorkshire pig (YP) is an imported breed and is characterized by high growth rate, elevated lean meat percentage and inferior meat quality [14, 15]. Several previous researches in large domestic animals also demonstrated that the profile of gut microbiota showed large distinction between breeds [16–18]. Thus, we speculated that there are huge differences in the gut microbiota compositions among TP, YP and RP.

Recently, there has been a considerable increase in the study of gut commensal flora and local host-microbe interactions. Host-microbe interactions occur initially along the surfaces of mucosa, and the intestinal mucosa contributes the largest surface area within the body, which separates the lumenal digesta from the internal milieu through a single layer of epithelium and represents a major surface for microbial colonization [19]. The overall balance in the composition of the gut microbial communities is important in ensuring homeostasis at the intestinal mucosa. Comparisons of germ free animals and conventional raised animals reveal the vital role of gut microbiota in the structural and functional development of the gastrointestinal tract [20–23]. A better intestinal morphology is beneficial for nutrients digestion and absorption. Moreover, gut barrier integrity can be improved by the preferable capacity of digestion and absorption in host [1]. Currently, fecal microbiota transplant (FMT) technology and the human flora-associated (HFA) animal model based on germ-free animals are commonly used in the research of the relationship between gut microbes and the human phenotype [24, 25]. Studies of transplanting fecal microbota from healthy human to those with various diseases (such as inflammatory bowel disease, clostridium difficile enterocolitis, metabolic phenotype and disease, immune disorder) have indicated that it is crucial to recover the normal microbial composition of the host during treatment for these diseases [26–29]. However, whether the gut properties are transmissible among animal species via fecal microbiota transplantation remains unclear. A study conducted by Zhang (2008) found that the allometry value of small intestine in Tibetan pig was smaller than that in foreign breeds [30]. Therefore, we hypothesized that the differences in gut characteristics among pig breeds can be transferred from the pig donors to germ-free (GF) mouse recipient.

The objective of present study was conducted to explore differences in the gut microbiota profiles and phenotypes in terms of intestinal morphology, digestive and absorptive capacity and barrier among TP, YP and RP and their mice recipients, and to determine whether the differences in the gut characteristics are transmissible via fecal microbiota transplantation. Thus, the relative achievements of this study will provide evidences for further understanding interactions among microbiota and host gut characteristics, interpreting the possible mechanism of gut microbiota regulating host gut development, and illuminating the possible way manipulating gut health by utilizing gut microbiota as the key target.

## Methods

### Animal experiment

The 5 Yorkshire pigs, 5 Tibetan pigs and 5 Rongchang pigs (12 weeks of age) were used in this experiment as fecal donors. All pigs were provided by reservation farms for these three pig breeds. TP, YP and RP were housed separately in individual metabolic cages in three environmentally controlled rooms on our experimental farm for 8 weeks until sacrifice, in which pigs were allowed *ad libitum* access to water and diet. A corn-soybean diet was formulated according to NRC (2012) requirements and Chinese feeding standards for local pigs (2004).

Germ-free BALB/C mice were provided by the Department of Laboratory Animal Science of the Third Military Medical University. A total of 24 1-day-old germ-free BALB/C mice, which were maintained in sterile Trexler plastic film isolators (Fengshi Laboratory Animal Equipment, China) and housed in polycarbonate cages on sterile wood chips at 22 ~ 24 °C at a relative air humidity of 45 ~ 55 % on a 12-h light–dark cycle, were used as recipients for fecal microbiota transplantation in this study. 1-day-old mice were breast fed by the germ free foster mice before weaning, and then were fed *ad libitum* with a chow diet sterilized by 60Co gamma radiation after they were weaned.

### Fecal microbiota transplantation and treatments

On the basis of the standard for donor identification and screening described by Hamilton et al., pigs used in the present study did not have diarrhea or other digestive disorders, never received medication before the study, and were fed a diet without antibiotics and probiotics for at least 2 month before feces collection [31]. The fresh feces of all the pigs were collected separately after 12-h fasting. In order to acquire representative fecal material for each breed, feces samples of each breed were mixed and then used as fecal inoculum. The remaining feces of each pig were stored at −80 °C until DNA extraction.

The stool suspension was prepared as described by Zeng et al. (2013) [32]. In brief, 1:9 (w/v) sterile prereduction phosphate buffer (0.1 mol/L, pH7.2) was added into the mixed fresh feces. The suspension was mixed and passed through 2.0, 1.0 and 0.5 mm stainless steel laboratory sieves to remove larger particles, and then stored at −80 °C until fecal transplantation.

Newborn germ-free mice in each treatment were infused by intragastric gavage with 0.05 mL fecal suspension of Tibetan, Yorkshire or Rongchang pigs, and 2 mL aliquots were spread on the fur of each germ-free foster mouse. These mice were maintained in the same manner as germ-free mice. Three treatment groups were: 1) Tibetan porcine flora-associated mice (TFM), 2) Yorkshine porcine flora-associated mice (YFM), and, 3) Rongchang porcine flora-associated mice (RFM). Each group contained 8 mice. The study lasted for 5 weeks.

### Sample collection of donors and recipients

Mouse pups were acquired by provoking defecation through slightly pushing in the lower abdomen using a moist cotton swab on week 5. All fecal samples were immediately stored at −80 °C until DNA extraction. All pigs were sacrificed at 20 weeks of age by using intravenously administrated dose of chlorpromazine hydrochloride (2 mg/kg body weight) as anesthetics. All mice were sacrificed at 5 weeks of age, and fasted overnight on the day before being killed by cervical dislocation. The length and weight of the small intestine and large intestine were measured, and the tissues of duodenum, jejunum and ileum were immediately isolated and preserved in 4 % paraformaldehyde solution. In addition, the tissues of duodenum, jejunum and ileum were immediately collected and stored at −80 °C.

### 16S rRNA amplicon sequencing

Total DNA was isolated using the QIAamp DNA stool Mini Kit (Qiagen, GmbH Hilden, Germany). The concentration and purity of extracted genomic DNA were measured using a NanoDrop ND-1000 Spectrophotometer (NanoDrop, Germany). The integrity of extracted genomic DNA was determined by electrophoresis on 1 % agarose gels. Sequencing and bioinformatics analysis were performed by BGI (Shenzhen, China). DNA library was prepared before high-throughput sequencing as previously described [33]. The resulting sequences were clustered to operational taxonomic units (OTU) using USEARCH drive5 at 97 % sequence identity. Relative abundance of each OTU was examined at different taxonomic levels.

### Intestinal index

The relative length, density and weight of intestine were calculated based on the formula shown as follows:

$$ \mathrm{Relative}\ \mathrm{length}\ \mathrm{of}\ \mathrm{intestine}\ \left(\mathrm{mm}/\mathrm{g}\right) = \mathrm{intestinal}\ \mathrm{length}/\mathrm{body}\ \mathrm{weight} $$

$$ \mathrm{Relative}\ \mathrm{density}\ \mathrm{of}\ \mathrm{intestine}\ \left(\mathrm{g}/\mathrm{cm}\right) = \mathrm{intestinal}\ \mathrm{weight}/\mathrm{intestinal}\ \mathrm{length} $$

$$ \mathrm{Relativeweightof}\ \mathrm{intestine}\ \left(\%\right) = \mathrm{intestinal}\ \mathrm{weight}/\mathrm{body}\ \mathrm{weight}\times 100 $$

### Histology of intestine

The duodenal, jejunal and ileal histomorphology were determined as described previously [34]. Briefly, following the fixing, the segment of the jejunum was excised, dehydrated and embedded in paraffin. Then, consecutive sections (5 μm) were cut and stained with haematoxylin and eosin. The villus height and crypt depth of the jejunal mucosa were determined by a single experimenter, blind to the pig breed and source of gut microbiota, and measured at 40 × magnification with an Olympus CK 40 microscope (Olympus Optical Company).

### Antioxidant capacity and the digestive and absorptive enzyme activities

The jejunal crude enzyme solution was prepared according to procedure previously described by Diao et al. (2015) [35]. The jejunum were collected and homogenized in 10 mmol/L ice-cold phosphate-buffered saline (m:v = 1:9). Homogenates were centrifuged at 2500 rpm at 4 °C for 10 min, and then the supernatant was used for subsequent analysis of the antioxidant capacity (methane dicarboxylic aldehyde (MDA), superoxide dismutase (SOD) and total antioxidant capacity (T-AOC)), and digestive and absorptive enzyme activities (protein content, amylase, lipase, trypsin, lactase, sucrase, maltase, alkaline phosphatase (AKP), Na^+^, K^+^-ATPase, Ca^+^, Mg^+^-ATPase and γ-glutamyltransferase (γ-GT)), which were measured by the commercial kits (Nanjing Jiancheng Institute of Bioengineering, Jiangsu, China) with UV–VIS Spectrophotometer (UV1100, MAPADA, Shanghai, China) according to the manufacturers’ protocols.

### Total RNA extraction, reverse transcription reaction and real-time quantitative PCR

Total RNA was isolated from the frozen duodenum, jejunum and ileum using the TRIzol reagent (TaKaRa Biotechnology (Dalian) Co., Ltd., Dalian, China) according to the manufacturer’s protocol. The synthesis of the first strand of cDNA of each sample was obtained by reverse transcription by RT Reagents (TaKaRa Biotechnology (Dalian) Co., Ltd., Dalian, China) according to the manufacturer’s instructions. The genes which were relative to cell differentiation (CDX2, caudal-related homeodomain transcription 2), intestinal development (EGF, epidermal growth factor; GLP-2, glucagon-like peptide-2; ANG4, angiogenin 4; IGF-1, insulin-like growth factor-1; IGF-1R, insulin-like growth factor-1 receptor), digestion and absorption (SGLT-1, sodium/glucose cotransporter 1; GLUT-2, glucose transporter type 2; ZNT1, zinc transporters-1; DMT1, divalent metal transporter-1; SLC_7_A_1_,solute carrier family 7), and intestinal barrier (MUC1, mucin 1; MUC2, mucin 2; REGIIIγ, regeneration protein IIIγ; Occludin; ZO-1, zonula occludens 1) can be detected by real-time quantitative PCR with the CFX96 Real-Time PCR Detection System (Bio-Rad Laboratories, Richmond, CA) as described by Zhao et al. (2014) [36]. The primers were synthesized commercially by Life Technologies Limited, which were listed in Additional file 1: Table S1.

### Statistical analysis

Data were analyzed by ANONA using the statistical program SAS 9.2 (SAS Inst. Inc., NC) where each pig or mouse was the statistical unit. All differences were considered significant at *P* < 0.05 and were considered a trend at 0.05 *≤ P* < 0.10. Principal coordinates analysis (PCoA) plots were produced using weighted UniFrac metrics. Plots were visualized using the R software (Packages ape). Log10-transformation was applied on the genus relative abundance data matrix for the heatmaps representation, which allowed visualizing similarities or differences between samples.

## Results

### 16S rRNA analysis of bacterial communities

**Fig. 1:**
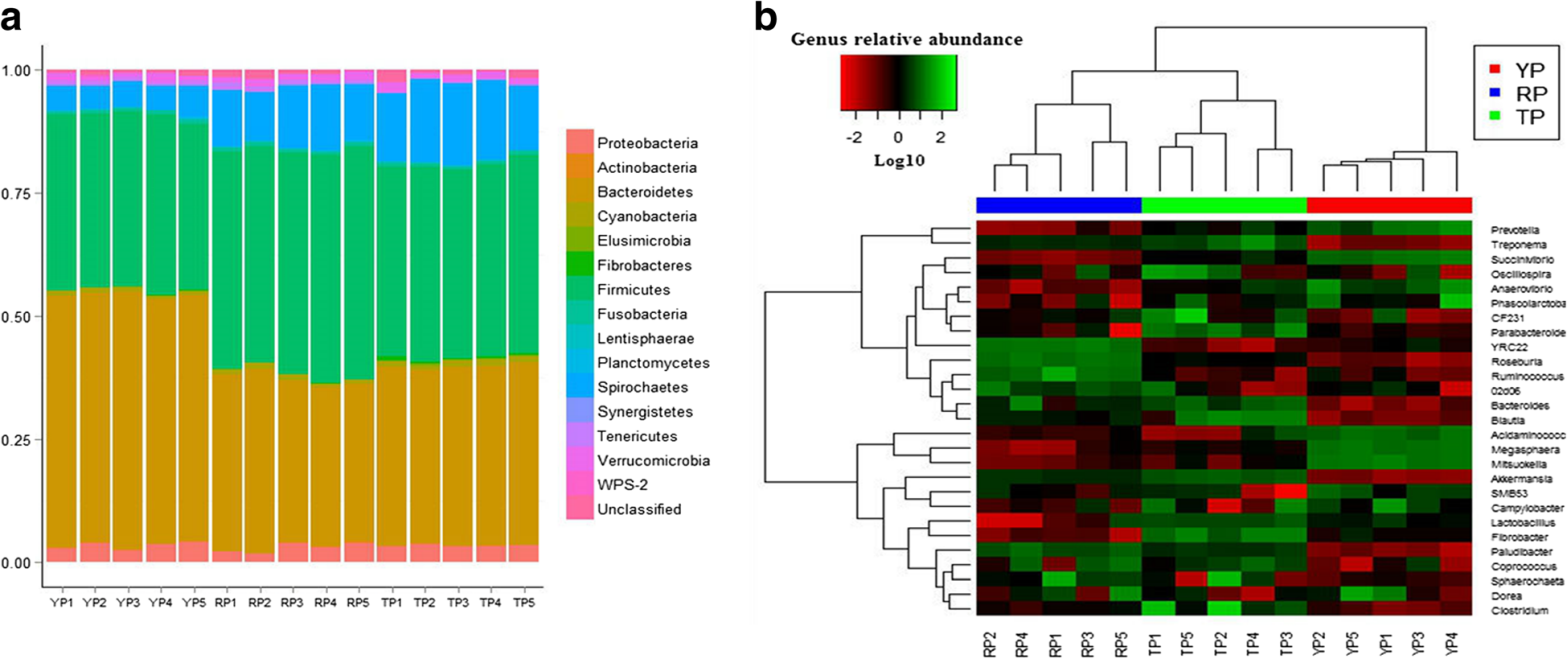
16S rRNA gene analysis reveals phyla and genus level differences in microbiota of TP, YP and RP. **a** Relative abundance of bacterial phyla present in TP, YP and RP. **b** Heatmap of log10-transformed abundance of selected genera (>0.1 % in at least one sample) for individual TP, YP and RP samples. Pigs with the highest and lowest bacterial levels are green and red, respectively. YP, Yorkshire pigs; TP, Tibetan pigs; RP, Rongchang pigs

Fecal samples of all pigs were dominated by *Bacteroidetes*, *Firmicutes*, *Spirochaetes* and *Proteobacteria*. A total of 15 phyla were shared by three pig breeds (Fig. 1a). Fourteen phyla (>0.1 % in at least 1 sample) were chosen for significance analysis. Compared with YP, RP and TP had higher proportions of bacteria in *Firmicutes* and *Spirochaetes* and lower proportion of bacteria in *Bacteroidetes* (*P* < 0.05). Compared with RP, TP had higher proportions of bacteria in *Spirochaetes* and lower proportion of bacteria in *Firmicutes* (*P* < 0.05). Compared with TP, YP and RP had higher proportions of bacteria in *Tenericutes* and lower proportion of bacteria in *Elusimicrobia* and *Fibrobacteres* (*P* < 0.05) (Additional file 1: Table S2). All mice fecal samples were dominated by four phyla: *Bacteroidetes*, *Firmicutes*, *Proteobacteria* and *Fusobacteria*. The results shown in Fig. 3a described the phylotype distribution at the phyla level for mice recipients, and specific microbiota phyla present in pig donors were also detected in mice recipients. Phyla differences were replicated, higher proportions of *Firmicutes* and lower proportion of *Bacteroidetes* in RFM and TFM versus YFM, and higher proportions of *Firmicutes* and lower proportion of *Bacteroidetes* in TFM versus RFM (*P* < 0.05). In addition, TFM and YFM exhibited a higher proportion of *Proteobacteria* and a lower proportion of *Fusobacteria* than RFM, and TFM exhibited higher proportion of *Actinobacteria* and *Spirochaetes* than RFM and YFM (*P* < 0.05) (Additional file 1: Table S4).

**Fig. 2:**
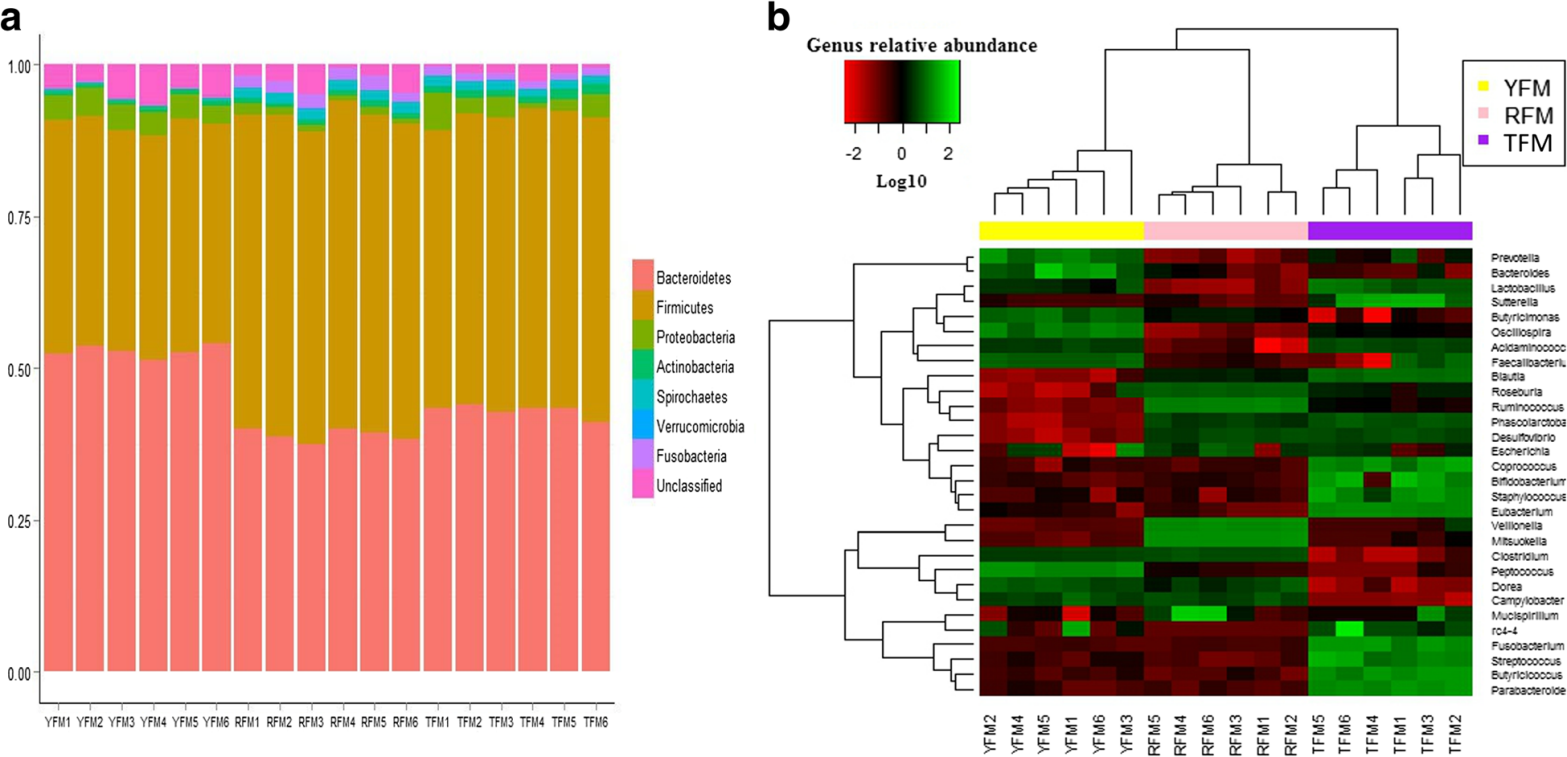
16S rRNA gene analysis reveals phyla and genus level differences in microbiota of TFM, YFM and RFM. **a** Relative abundance of bacterial phyla present in TFM, YFM and RFM. **b** Heatmap of log10-transformed abundance of all observed genera for individual TFM, YFM and RFM samples. Mice with the highest and lowest bacterial levels are green and red, respectively. TFM, Tibetan porcine flora-associated mice; TFM, Yorkshire porcine flora-associated mice; RFM, Rongchang porcine flora-associated mice

Figure 1b represented a heatmap showing the abundances of selected genera (>0.1 % in at least 1 sample) across all pig samples. It clearly showed that there were apparent differences in genus distribution among YP, TP and RP fecal microbiota. The proportions of *YRC22*, *Ruminococcus*, *Paludibacter* and *Roseburia* were higher in RP than YP and TP, whereas the proportions of bacteria in *Prevotella*, *Succinivibrio*, *Anaerovibrio*, *Acidaminococcus*, *Megasphaera*, *SMB53* and *Mitsuokella* were higher in YP than TP and RP. In addition, the proportions of *Treponema*, *CF231*, *Lactobacillus*, *Parabacteroides*, *Clostridium*, *Blautia*, *Fibrobacter* and *Akkermansia* were higher in TP than YP and RP (*P* < 0.05) (Additional file 1: Table S3). Likewise, bacterial genera distribution differed between TFM, YFM and RFM and several genera (*Bacteroides*, *Blautia*, *Lactobacillus*, *Parabacteroides*, *Prevotella*, *Roseburia* and *Ruminococcus*) differences existing in pig donors were conserved in mice recipients (*P* < 0.05) (Fig. 2b, Additional file 1: Table S5). In addition, TFM had higher proportions of bacteria in *Acidaminococcus*, *Bifidobacterium*, *Butyricicoccus*, *Coprococcus*, *Desulfovibrio*, *Eubacterium*, *Fusobacterium*, *Staphylococcus*, *Streptococcus* and *Sutterella* than YFM and RFM, and YFM and RFM had higher proportions of bacteria in *Butyricimonas*, *Clostridium*, *Faecalibacterium*, *Oscillospira*, *Peptococcus* and *Phascolarctobacterium* than TFM, and RFM had higher proportions of bacteria in *Mitsuokella* and *Veillonella* than YFM and TFM (Additional file 1: Table S5, *P* < 0.05).

**Fig. 3:**
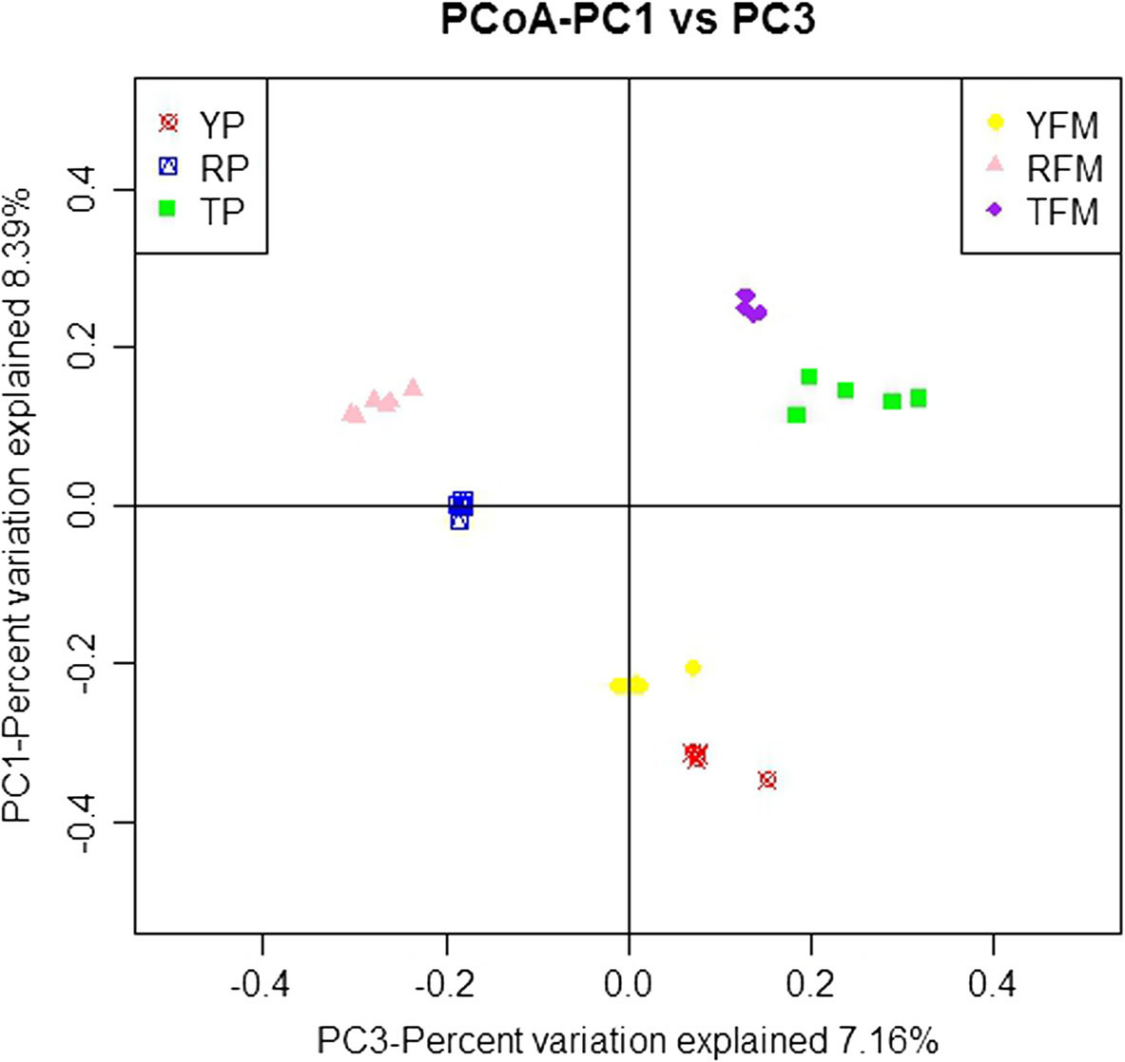
Comparison of the gut microbiota composition among 6 groups. Principal coordinate analysis to visualize the weighted UniFrac distances of fecal samples from individual pigs and mice. YP, Yorkshire pigs; TP, Tibetan pigs; RP, Rongchang pigs, TFM, Tibetan porcine flora-associated mice; TFM, Yorkshire porcine flora-associated mice; RFM, Rongchang porcine flora-associated mice

Moreover, the principal coordinates analysis was carried out to measure the extent of the similarity between microbiota communities. The fecal microbiota from YP, TP and RP could be divided into three different clusters and could be separated clearly by PCoA (Fig. 3). The community structures observed in YFM, TFM and RFM samples were significantly different from each other (Fig. 3). PCoA plots showed that YFM, TFM and RFM samples formed three different clusters, and fecal samples of mice recipients formed a cluster close to its donor fecal samples. Therefore, the bacterial microbiota showed marked divergence between YP, TP and RP, and the mice recipients shared a high similarity in the gut microbiota with their pig donors.

### The three groups of pig donors and their mice recipients exhibited different intestinal index and morphology

#### Intestinal index and morphology

The relative weight of large intestine and total intestine in TP and RP were higher than that in YP (*P* < 0.05) (Table 1). RP had the higher relative density of large intestine (*P* < 0.05) and total intestine (*P* = 0.059) compared with TP and YP. The length of intestine (small, large and total) in TP was higher than that in YP and RP (*P* < 0.05). Likewise, the relative weight of small intestine and total intestine in RFM and TFM were higher than that in YFM (*P* < 0.05) (Table 2). The relative density of intestine (small, large and total) in RFM was higher than that in YFM (*P* < 0.05). Compared with RFM, YFM had lower length of small intestine (*P* = 0.094), large intestine (*P* = 0.069) and total intestine (*P* < 0.05).

**Table 1:** Effect of genotype on intestinal index and morphology in pigs

Items	TP	YP	RP	SEM	*P*-value
Relative length of SI (mm/g)	2.341^a^	1.744^b^	1.671^b^	0.099	0.003
Relative length of LI (mm/g)	0.942^a^	0.497^b^	0.552^b^	0.050	0.001
Relative length of I (mm/g)	3.283^a^	2.241^b^	2.223^b^	0.143	0.001
Relative density of SI (g/cm)	0.756	0.889	0.940	0.079	0.290
Relative density of LI (g/cm)	3.225^b^	3.158^b^	4.550^a^	0.213	0.003
Relative density of I (g/cm)	1.456	1.385	1.841	0.121	0.059
Relative weight of SI (%)	1.731	1.552	1.541	0.084	0.281
Relative weight of LI (%)	2.987^a^	1.555^b^	2.506^a^	0.166	0.001
Relative weight of I (%)	4.718^a^	3.107^b^	4.047^a^	0.215	0.002
Duodenum					
Villus height (μm)	722.380^a^	534.540^b^	521.160^b^	51.073	0.042
Crypt depth (μm)	395.980^a^	288.010^ab^	255.270^b^	32.871	0.039
Villus height: crypt depth	1.919	1.906	2.162	0.331	0.831
Jejunum					
Villus height (μm)	531.280^a^	433.240^b^	437.910^b^	26.658	0.050
Crypt depth (μm)	280.490^ab^	288.130^a^	204.470^b^	19.424	0.029
Villus height: crypt depth	1.987^ab^	1.565^b^	2.146^a^	0.140	0.046
Ileum					
Villus height (μm)	476.703^a^	457.320^a^	255.050^b^	30.871	0.002
Crypt depth (μm)	247.102^a^	303.920^a^	142.950^b^	25.648	0.006
Villus height: crypt depth	1.990	1.544	1.876	0.188	0.275

*TP* Tibetan pig; *YP* Yorkshire pig; *RP* Rongchang pig; *SI* small intestine; *LI*, large intestine; *I* intestine

Relative length of intestine (mm/g) = intestinal length/body weight

Relative density of intestine (g/cm) = intestinal weight/ intestinal length

Relative weight of intestine (%) = intestinal weight/body weight

^a-b^Within a row, means without a common superscript differ (*P* < 0.05)

**Table 2:** Effect of gut flora source on intestinal index and morphology in mice

Items	TFM	YFM	RFM	SEM	*P*-value
Relative length of SI (cm/g)	2.015	1.908	1.813	0.053	0.094
Relative length of LI (cm/g)	0.510	0.490	0.405	0.027	0.069
Relative length of I (cm/g)	2.528^a^	2.395^ab^	2.218^b^	0.051	0.014
Relative density of SI (g/cm)	0.027^ab^	0.023^b^	0.030^a^	0.002	0.008
Relative density of LI (g/cm)	0.079^b^	0.078^b^	0.100^a^	0.001	0.049
Relative density of I (g/cm)	0.037^ab^	0.034^b^	0.042^a^	0.002	0.008
Relative weight of SI (%)	5.267^a^	4.336^b^	5.330^a^	0.189	0.012
Relative weight of LI (%)	3.987	3.812	3.902	0.068	0.371
Relative weight of I (%)	9.254^a^	8.148^b^	9.231^a^	0.216	0.036
Duodenum					
Villus height (μm)	444.201^a^	370.622^b^	367.560^b^	16.610	0.029
Crypt depth (μm)	94.481	89.989	85.782	9.660	0.822
Villus height: crypt depth	5.028	4.241	4.338	9.660	0.619
Jejunum					
Villus height, μm	477.101^a^	337.110^b^	348.704^b^	23.583	0.010
Crypt depth, μm	86.190	73.722	75.781	3.084	0.059
Villus height: crypt depth	1.987^ab^	1.565^b^	2.146^a^	0.140	0.046
Ileum					
Villus height (μm)	165.586	149.821	145.269	17.501	0.704
Crypt depth (μm)	51.262	49.183	49.960	7.991	0.982
Villus height: crypt depth	3.251	3.051	3.010	0.242	0.761

*TFM* Tibetan porcine flora-associated mice; *YFM* Yorkshire porcine flora-associated mice; *RFM* Rongchang porcine flora-associated mice; *SI* small intestine; *LI* large intestine; *I* intestine

^a-b^Within a row, means without a common superscript differ (*P* < 0.05)

The villus height of duodenum and jejunum in TP was increased compared with YP and RP, and the villus height of ileum in TP and YP was higher than that in RP (*P* < 0.05) (Table 1). TP had higher crypt depth of duodenum than RP, YP had higher crypt depth of jejunum than RP, and TP and YP had higher crypt depth of ileum than RP (*P* < 0.05). The villus height: crypt depth of jejunum in RP was higher than that in YP (*P* < 0.05). Similarly, the villus height of duodenum and jejunum in TFM was higher than that in other two groups (*P* < 0.05) (Table 2). Moreover, there was a tendency towards increased jejunal crypt depth in TFM compared to YFM and RFM (*P* = 0.059).

#### Intestinal cell proliferation and differentiation

**Fig. 4:**
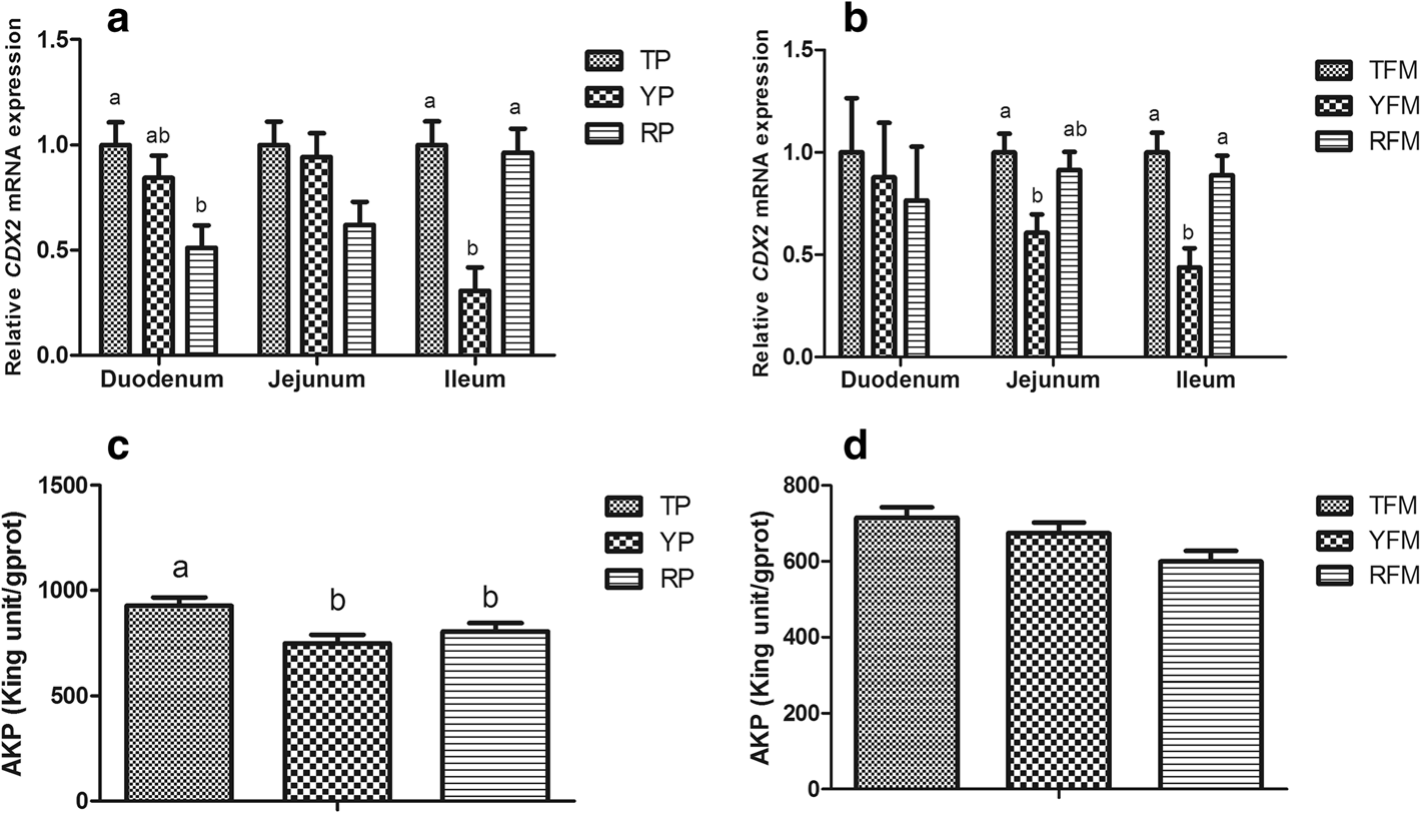
Intestinal cell proliferation and differentiation. **a** Relative amounts of CDX2 mRNAs in duodenum, jejunum and ileum of TP, YP and RP. **b** Relative amounts of CDX2 mRNAs in duodenum, jejunum and ileum of TFM, YFM and RFM. **c** AKP (alkaline phosphatase) concentration in jejunum of TP, YP and RP. **d** AKP concentration in jejunum of TFM, YFM and RFM. Results are presented as mean ± SEM. a-b means without a common superscript differ (*P* < 0.05). YP, Yorkshire pigs; TP, Tibetan pigs; RP, Rongchang pigs, TFM, Tibetan porcine flora-associated mice; TFM, Yorkshire porcine flora-associated mice; RFM, Rongchang porcine flora-associated mice

As shown in Fig. 4, the relative CDX2 mRNA expression of duodenum in TP was higher than that in RP, and TP and RP had higher relative CDX2 mRNA expression in ileum compared with YP (*P* < 0.05). TP exhibited increasing jejunal AKP activity compared with YP and RP (*P* < 0.05). Likewise, compared with TFM and RFM, YFM had lower relative CDX2 mRNA expression in ileum (*P* < 0.05). In addition, the jejunal AKP activity in TP was higher than that in RP (*P* = 0.058).

#### The relative mRNA expression of intestinal development-related genes

**Fig. 5:**
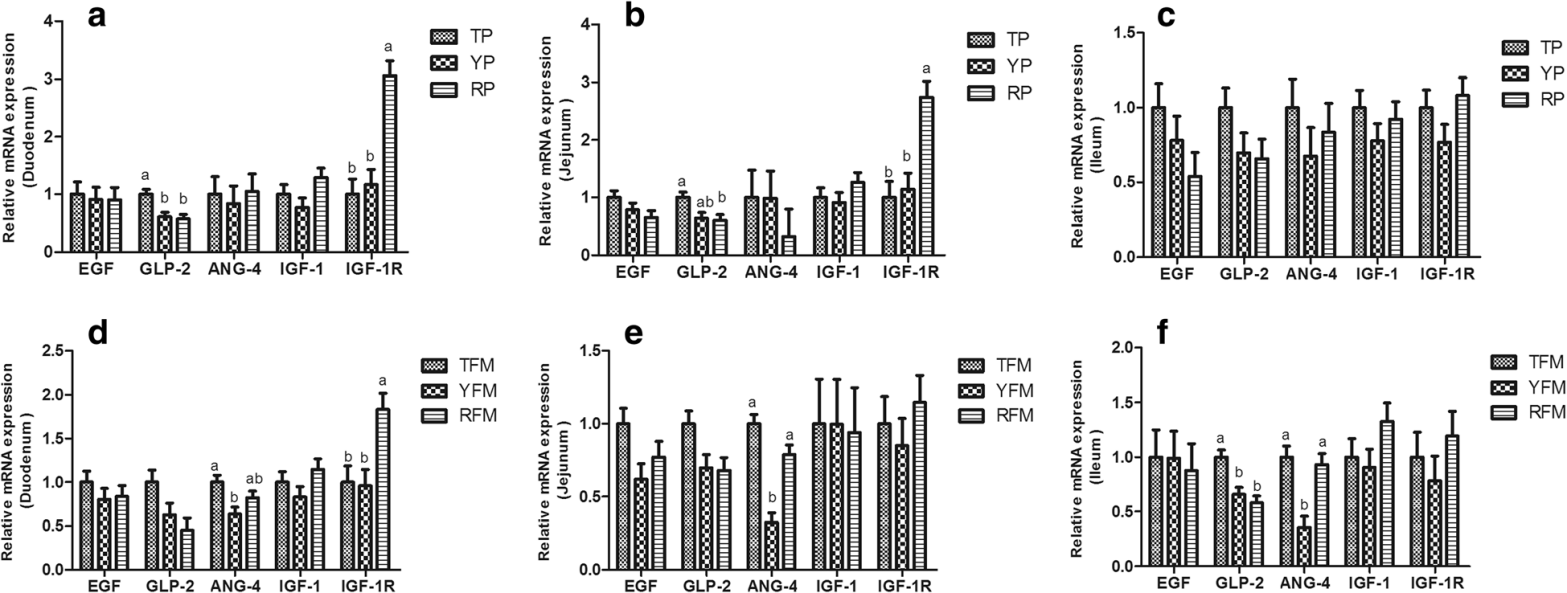
The relative mRNA expression of intestinal development-related gene. **a**–**c** Relative amounts of EGF, GLP-2, ANG-4, IGF-1 and IGF-1R mRNAs in duodenum, jejunum and ileum of TP, YP and RP. **d**–**f** Relative amounts of EGF, GLP-2, ANG-4, IGF-1 and IGF-1R mRNAs in duodenum, jejunum and ileum of TFM, YFM and RFM. Results are presented as mean ± SEM. a-b means without a common superscript differ (*P* < 0.05). YP, Yorkshire pigs; TP, Tibetan pigs; RP, Rongchang pigs, TFM, Tibetan porcine flora-associated mice; TFM, Yorkshire porcine flora-associated mice; RFM, Rongchang porcine flora-associated mice

As shown in Fig. 5, TP had higher relative GLP-2 mRNA expression of duodenum compared with RP and YP, and had higher relative GLP-2 mRNA expression of jejunum compared with RP (*P* < 0.05). The relative IGF-1R mRNA expression of duodenum and jejunum in RP was higher than that in TP and YP (*P* < 0.05). Similarly, the relative GLP-2 mRNA expression of duodenum (*P* = 0.072), jejunum (*P* = 0.073), and ileum (*P* < 0.05) in TFM was higher than that in YFM and RFM. RFM expressed higher relative IGF-1R mRNA expression of duodenum than that in TFM and YFM (*P* < 0.05). Interestingly, the relative ANG4 mRNA expression of jejunum and ileum in TFM and RFM were higher than that in YFM (*P* < 0.05).

### The digestive and absorptive ability was different among the three groups of pig donors and their mice recipients

#### Digestive and absorptive enzyme activity in jejunum

Compared with TP and RP, YP had higher lactase (*P* = 0.050) and maltase (*P* < 0.05) activities (Table 3), and YP and RP had higher sucrase activity than TP (*P* = 0.084). However, the amylase of jejunum in TP was higher than that in YP and RP, and the activity of jejunal trypsin in TP was higher than that in RP (*P* < 0.05). Moreover, the activities of jejunal Na^+^, K^+^-ATPase, Ca^+^, Mg^+^-ATPase and γ-GT in TP was higher than that in YP and RP (*P* < 0.05). Similarly, compared with TFM and RFM, YFM exhibited decreasing total protein (*P* = 0.067), and increasing activities of sucrase (*P* < 0.05) and maltase (*P* < 0.05) in jejunum (Table 4). TFM exhibited higher activity jejunal γ-GT than YFM and RFM (*P* < 0.05).

**Table 3:** Effect of genotype on digestive and absorptive enzyme activity in jejunum of pigs

Items	TP	YP	RP	SEM	*P*-value
Total protein (mgprot/ml)	0.722	0.698	0.738	0.016	0.262
Lactase (U/mgprot)	44.732^b^	87.812^a^	56.107^b^	10.748	0.050
Sucrase (U/mgprot)	195.548	263.061	266.161	21.879	0.084
Maltase (U/mgprot)	296.851^b^	429.180^a^	329.132^ab^	28.179	0.026
Amylase (U/mgprot)	127.980^a^	69.680^b^	60.450^b^	11.178	0.005
Lipase (U/gprot)	108.140	103.930	61.470	16.255	0.141
Trypsin (U/mgprot)	1510.600^a^	1179.100^ab^	833.200^b^	125.913	0.016
Na^+^, K^+^-ATPase (μmolPi/mgprot/hour)	7.519^a^	5.592^b^	5.215^b^	0.383	0.006
Ca^+^, Mg^+^-ATPase (μmolPi/mgprot/hour)	7.494^a^	6.709^a^	3.896^b^	0.630	0.009
γ-GT(U/gpro)	57.052^a^	46.181^b^	49.670^ab^	2.223	0.023

*TP* Tibetan pig; *YP* Yorkshire pig; *RP* Rongchang pig

^a-b^Within a row, means without a common superscript differ (*P* < 0.05)

**Table 4:** Effect of gut flora source on digestive and absorptive enzyme activities in jejunum of mice

Items	TFM	YFM	RFM	SEM	*P*-value
Total protein(mgprot/ml)	1.266	1.156	1.237	0.027	0.067
Lactase (U/mgprot)	65.662	74.228	67.777	17.755	0.923
Sucrase (U/mgprot)	0.758^b^	16.381^a^	0.932^b^	2.676	0.009
Maltase (U/mgprot)	120.710^b^	157.626^a^	119.301^b^	6.714	0.011
Amylase (U/mgprot)	0.445	0.419	0.335	0.058	0.429
Lipase (U/gprot)	37.840	32.930	22.240	7.157	0.354
Trypsin (U/mgprot)	60.930	58.070	52.780	10.796	0.867
Na^+^, K^+^-ATPase (μmolPi/mgprot/hour)	5.085	4.847	4.231	0.281	0.166
Ca^+^, Mg^+^-ATPase (μmolPi/mgprot/hour)	4.653	4.751	4.121	0.437	0.577
γ-GT(U/gpro)	34.102^a^	26.514^b^	28.399^b^	1.014	0.005

*TFM* Tibetan porcine flora-associated mice; *YFM* Yorkshire porcine flora-associated mice; *RFM* Rongchang porcine flora-associated mice

^a-b^Within a row, means without a common superscript differ (*P* < 0.05)

**Table 5:** Effect of genotype on jejunal antioxidant capacity in pigs

Items	TP	YP	RP	SEM	*P*-value
MDA (nmol/mgprot)	0.886	1.342	1.182	0.122	0.078
T-AOC (U/mgprot)	0.148	0.130	0.123	0.008	0.145
SOD (U/mgprot)	117.950	116.750	113.290	8.018	0.914

*TP* Tibetan pig; *YP* Yorkshire pig; *RP* Rongchang pig

^a-b^Within a row, means without a common superscript differ (*P* < 0.05)

#### Digestion and absorption-related genes in small intestine

**Fig. 6:**
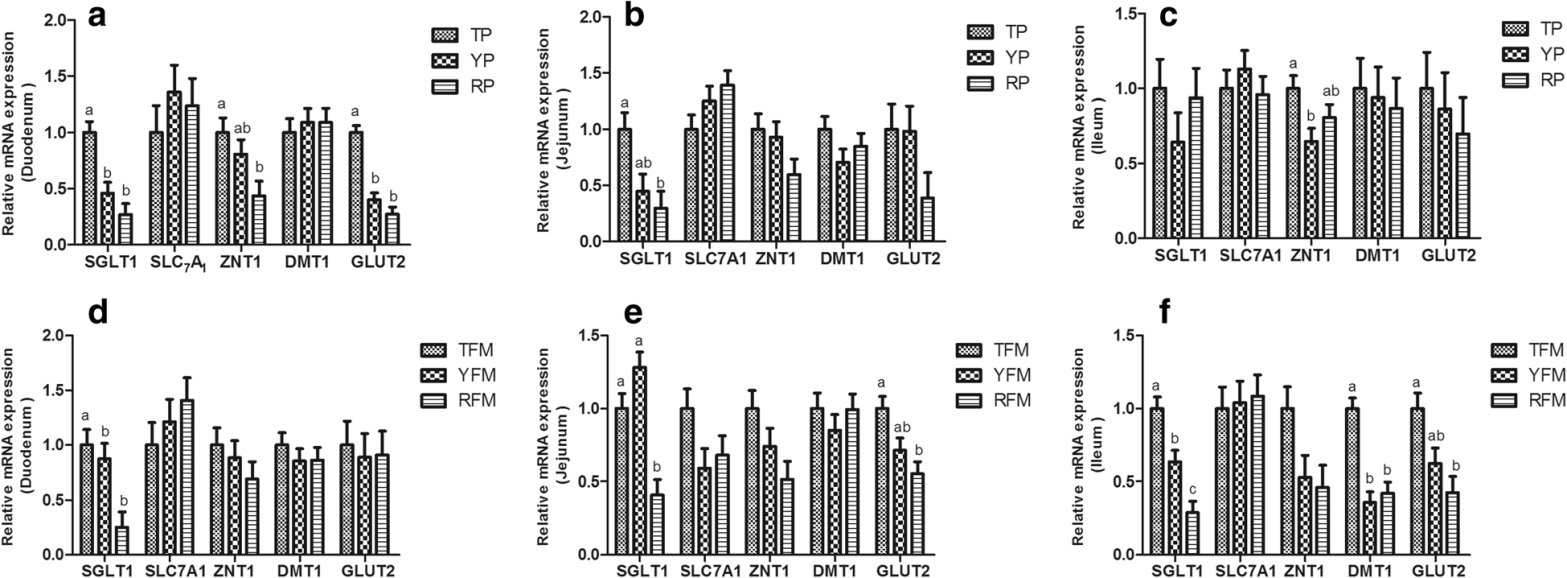
The relative mRNA expression of digestion and absorption-related genes in small intestine. **a**–**c** Relative amounts of SGLT1, SLC7A1, ZNT1, DMT1 and GLUT2 mRNAs in duodenum, jejunum and ileum of TP, YP and RP. **d**–**f** Relative amounts of SGLT1, SLC7A1, ZNT1, DMT1 and GLUT2 mRNAs in duodenum, jejunum and ileum of TFM, YFM and RFM. Results are presented as mean ± SEM. a-b means without a common superscript differ (*P* < 0.05). YP, Yorkshire pigs; TP, Tibetan pigs; RP, Rongchang pigs, TFM, Tibetan porcine flora-associated mice; TFM, Yorkshire porcine flora-associated mice; RFM, Rongchang porcine flora-associated mice

As shown in Fig. 6, the relative SGLT1 and GLUT2 mRNA expressions of duodenum in TP were higher than those in YP and RP (*P* < 0.05). TP had higher relative ZNT1 mRNA expression of duodenum and relative SGLT1 mRNA expression of jejunum compared with RP (*P* < 0.05). The relative ZNT1 mRNA expression of ileum in TP was higher than that in YP (*P* < 0.05). Similarly, TFM exhibited increasing relative SGLT1 mRNA expression of duodenum and ileum (*P* < 0.05), relative ZNT1 mRNA expression of jejunum and ileum (*P* = 0.083), and relative DMT1 mRNA expression of ileum (*P* < 0.05) compared with YFM and RFM. Moreover, the relative SGLT1 mRNA expression of jejunum in TFM and YFM was higher than that in RFM, and the relative GLUT2 mRNA expression of jejunum and ileum in TFM was higher than that in RFM (*P* < 0.05).

**Table 6:** Effect of gut flora source on jejunal antioxidant capacity in mice

Items	TFM	YFM	RFM	SEM	*P*-value
MDA (nmol/mgprot)	0.584	0.709	0.570	0.041	0.098
T-AOC (U/mgprot)	1.867	1.448	1.329	0.138	0.073
SOD (U/mgprot)	92.140	96.840	95.470	7.685	0.907

*TFM* Tibetan porcine flora-associated mice; *YFM*Yorkshire porcine flora-associated mice; *RFM*Rongchang porcine flora-associated mice

^a-b^Within a row, means without a common superscript differ (*P* < 0.05)

### Tibetan pigs derived microbiota improved gut barrier in mice recipients

#### Jejunal antioxidant capacity

The concentration of MDA in YP was higher than that in TP and RP (*P* = 0.078) (Table 5). Likewise, compared with TFM and RFM, YFM exhibited increased MDA concentration in jejunum (*P* = 0.098) (Table 6). In addition, TFM had higher T-AOC activity compared with YFM and RFM (*P* = 0.073).

#### The relative mRNA expression of intestinal barrier-related genes

**Fig. 7:**
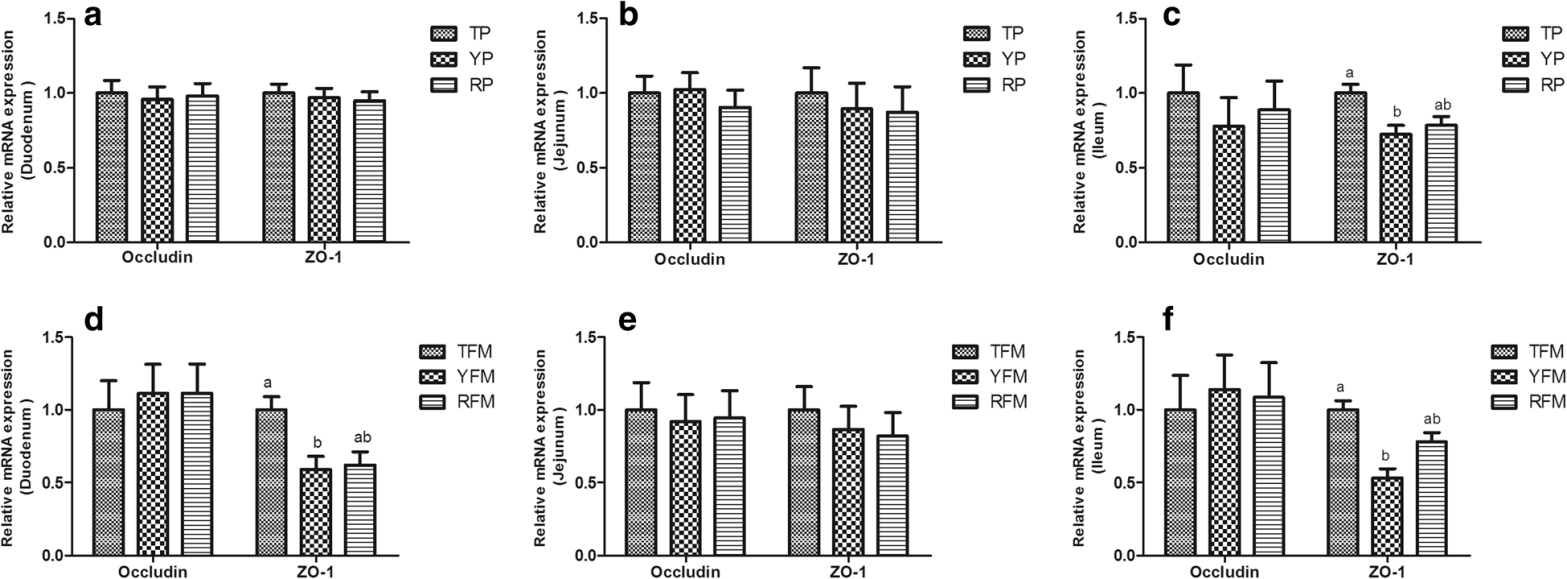
The relative mRNA expression of tight junction-related genes in small intestine. **a**–**c** Relative amounts of Occludin and ZO-1 mRNAs in duodenum, jejunum and ileum of TP, YP and RP. **d**–**f** Relative amounts of Occludin and ZO-1 mRNAs in duodenum, jejunum and ileum of TFM, YFM and RFM. Results are presented as mean ± SEM. a-b means without a common superscript differ (*P* < 0.05). YP, Yorkshire pigs; TP, Tibetan pigs; RP, Rongchang pigs, TFM, Tibetan porcine flora-associated mice; TFM, Yorkshire porcine flora-associated mice; RFM, Rongchang porcine flora-associated mice

**Fig. 8:**
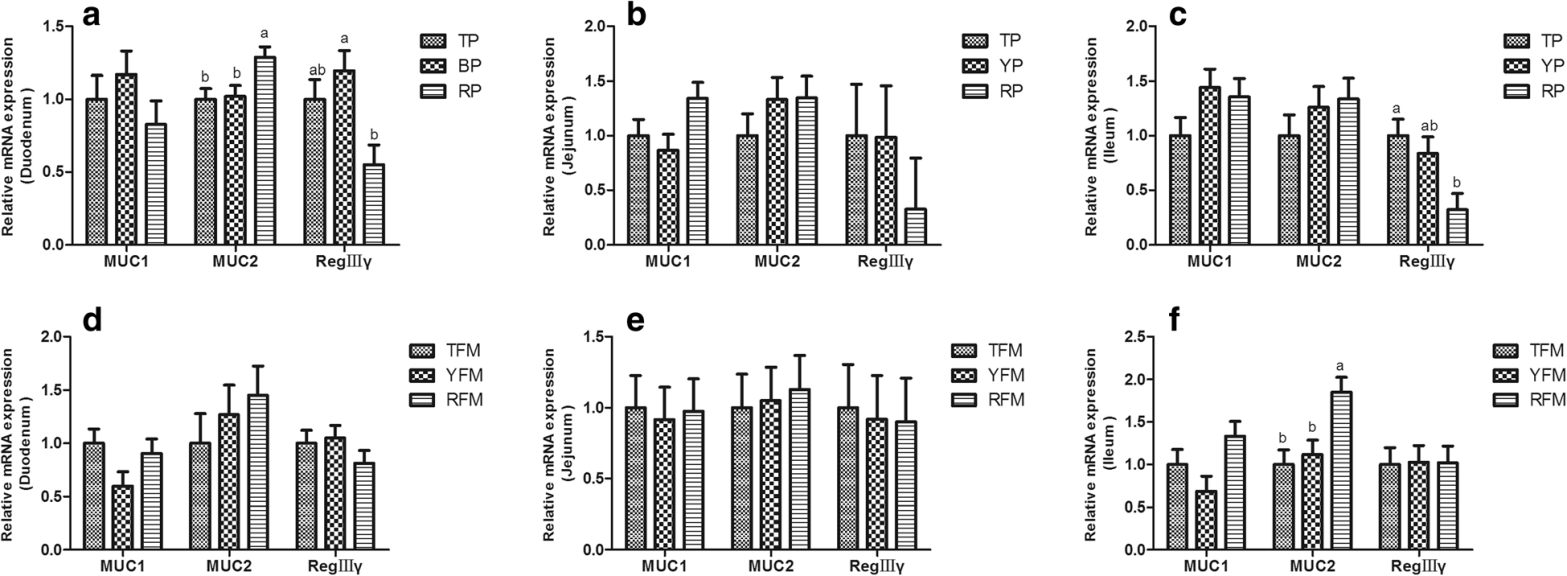
The relative mRNA expression of mucin and RegIIIγ in small intestine. **a**–**c** Relative amounts of MUC1, MUC2 and RegIIIγ mRNAs in duodenum, jejunum and ileum of TP, YP and RP. **d**–**f** Relative amounts of MUC1, MUC2 and RegIIIγ mRNAs in duodenum, jejunum and ileum of TFM, YFM and RFM. Results are presented as mean ± SEM. a-b means without a common superscript differ (*P* < 0.05). YP, Yorkshire pigs; TP, Tibetan pigs; RP, Rongchang pigs, TFM, Tibetan porcine flora-associated mice; TFM, Yorkshire porcine flora-associated mice; RFM, Rongchang porcine flora-associated mice

*As *shown in Fig. 7, the relative ZO-1 mRNA expression of ileum in TP was higher than that in YP (*P* < 0.05). RP had higher relative MUC2 mRNA expression of duodenum compared with TP (*P* < 0.05, Fig. 8). The relative RegIIIγ mRNA expression of duodenum in TP and YP, and that of jejunum in TP were higher than that in PR (*P* < 0.05). Similarly, the relative ZO-1 mRNA expression of duodenum and ileum in TFM was higher than that in YFM (*P* < 0.05, Fig. 7). Likewise, RFM increased the relative MUC2 mRNA expression of ileum compared with TFM (*P* < 0.05, Fig. 8).

## Discussion

Gut microbiota has been recognized as a strong determinant factor of host physiology, especially its critical role in host gut development [19]. The causal relationship between gut microbiota and host phenotypes has been widely studied via fecal microbiota transplantation. It has been shown that inflammatory bowel disease could be cured by transplanting fecal microbota from healthy human [28]. Previous studies indicated that the gut of individuals and animals with different genotype or phenotype may harbor distinct microbiota [16, 17, 37]. However, there is not enough evidence to indicate the relationship between gut microbiota and gut phenotype features. Here, we demonstrated that different pig breeds exhibit a distinct gut microbial profile. Moreover, we found that transfering porcine microbiota to mice recipients can replicate the gut characteristics of the pig donors.

16S rRNA gene sequencing in donor pigs and recipient mice showed that the fecal microbiota composition at the phylum and genus levels were widely different in TP, YP and RP, and so were the same phenomena in TFM, YFM and RFM. More specifically, lower *Firmicutes* and higher *Bacteroidetes* levels were observed in YP and YFM, whereas higher levels of bacteria from *Elusimicrobia*, *Fibrobacteres* and *Spirochaetes* were observed in TP, and a higher level of *Spirochaetes* was observed in TFM. Studies comparing the gut microbiota between obese and lean animals showed that lower *Firmicutes* and higher *Bacteroidetes* levels were associated with lean phenotype [18, 38]. As is known to us, YP is an imported breed and is characterized by high body lean mass, which is consistent with our study. *Spirochaetes* is capable of degrading polymers, such as xylan, pectin and arabinogalactan, and is found to be positively correlated with the apparent hemicellulose digestibility of pigs [39–41]. *Elusimicrobia* is the intracellular symbiont of termite gut flagellates, *Fibrobacteres* is an important phylum of cellulose-degrading bacteria, and both of them are capable of degrading fiber [42, 43]. TP were recognized to be more adaptable to poor dietary conditions than foreign pig breeds [30], and this may be attributed to higher *spirochaetes*, *Fibrobacteres* and *Elusimicrobia* proportion in gut microbiota. In addition, for the genus level, a higher proportion of *Prevotella* and a lower proportion of *Ruminococcus* were found in healthy weight adolescents compared with obese humans [44, 45], which is similar to our study, a higher proportion of *Prevotella* and lower proportion of *Ruminococcus* were found in YP and YFM. *Roseburia* and *Blautia* are major bacteria that produce butyrate and acetic acid respectively [46, 47]. In our study, TP and TFM exhibited increased abundance of *Roseburia* and *Blautia* compared with YP and YFM, which is beneficial to gut health. Moreover, *Lactobacillus* and *Parabacteroides* are found to cure enteritis [48, 49]. In the present study, TP and TFM had higher proportion of *Lactobacillus* and *Parabacteroides*, and this may be attributed to better gut characteristics in TP.

Previous studies indicated that gut communities in the same phenotype individuals were similar to each other [37, 50]. We also found that TP, YP and RP fecal microbiota could be divided into three separate clusters based on PCA through 16S rRNA gene sequencing. Previous studies have shown that human and rat microbiota can be transferred to GF mice with striking preservation of structure and diversity [51, 52], which was consistent with our study, recipient mice exhibited a high similarity in bacterial community structure with their corresponding pig donors. From the above, gut composition differs between TP, YP and RP, and the mice recipients share high similarity in the gut microbiota with their pig donors.

Long-term reproductive and environmental isolation may lead to specific profiles of organ development, digestive ability and nutrient deposition existing in different pig breeds during their adaptation and evolution [53, 54]. In the present study, the relative weight of total intestine in Chinese indigenous pig breeds (TP and RP) and their mice recipients (TFM and RFM) was significantly higher than that in YP and YFM, which was in agreement with the previous finding that indicated the lean-type pigs had a lower proportional weight of intestine than indigenous genotypes [55]. But many findings elucidated that pigs with leaner carcasses exhibited higher weights for small intestine and large intestine [54, 56]. An experiment with growth stages has shown that the development changes of relative viscera weight between lean pigs and fatter pigs was dissimilar in different growth stage [57, 58]. Thus, further research is clearly warranted. In this study, the intestine length did not differ between RP and YP, and the results were in accordance with previous findings [57], which indicated that the length of small intestine did not differ between foreign pig breeds and native pig breeds. However, the intestine length was higher in TP and TFM compared with YP and YFM in the current experiment. The differences of gut microbial ecology were observed between pig breeds, and changes in microbiota composition would affect the endogenous intestinotrophic proglucagon-derived peptide (GLP-2) production [38, 59]. It is well-known that IGF-1, EGF, GLP-2, and its receptor (GLP-2R) are important regulators of intestine length [60, 61], and ANG4 is a paneth cell granule protein shaping intestinal angiogenesis [62]. In this study, TP and TFM had highest GLP-2 mRNA expression among the three pig breeds and their mice recipients, and TFM had higher ANG4 mRNA expression compared with YFM, which are associated with higher intestine length in TP and TFM.

Previous reports confirmed that pig breeds produce variation in the structure of the small intestine [57]. In the present study, variations in small intestinal morphology were also observed among pig breeds and their mice recipients. Higher villus height of duodenum and jejunum was observed in TP and TFM compared with YP and RP, YFM and RFM, respectively. This is in accordance with data previously reported, villus surface was larger for Iberian pigs than Landrace × Large White pigs at 15 kg of BW [63]. As we know, the CDX2 and AKP are involved in intestinal cell proliferation and differentiation, which could contribute to the structure of the intestine [64, 65]. In our study, we found that TP displayed higher CDX2 mRNA expression in the ileum and AKP activity in the jejunum compared with YP, and TFM exhibited higher CDX2 mRNA expression in the ileum compared with YFM. These results are generally consistent with that of Rubio et al. (2010) who reported differences in AKP activity were found among pig breeds [63]. The abovementioned positive regulators must have a potent nutritional effect on the intestine growth and development, and these factors are differently expressed in the digestive tract among pig breeds and their mice recipients to account for differences in phenotype.

Small intestinal nutrient digestion and absorption was also affected by pig breeds. It has been observed that pig genotype (Iberian v. Landrace × Large White) exhibited different nitrogen retention and apparent total tract digestibility of nitrogen, energy and organic matter [57]. In a study of changes in small intestinal nutrient transport in Meishan pigs and Yorkshire pigs showed that different glucose, arginine, glutamine and threonine transportation in the small intestine were observed in the two pig breeds [53]. The ratio of *bacteroidetes* and *firmicutes* bacterial groups in the gut was increased in lean-type pigs which exhibited greater ability to absorb nutrients [66]. Consistent with previous work [63], which observed that Landrace × Large White pigs had higher activities of lactase, sucrase and maltase than Iberian pigs in the small intestine, our current study showed YP and YFM had elevated jejunal sucrase and maltase activities, which may have resulted from its higher ratio of *bacteroidetes* and *firmicutes* bacterial groups. Meanwhile, TP had higher jejunal amylase, and trypsin than YP or RP, which is generally consistent with the previous study of two pig breeds, total activities of lipase, trypsin and amylase at 49 d of age were 2.0, 1.5 and 5.0 fold higher, respectively, in Alentejano piglets than Large White piglets [67]. In our study, TP and TFM had higher jejunal γ-GT and relative SGLT1 mRNA expression, suggesting the differences of absorptive enzymes and transport carriers possibly affect nutrient digestion and absorption in different pig breeds. In addition, YP and YFM had highest MDA concentration in the jejunum among the three pig genotypes and their mice recipients in this study, and there is currently no available information on the difference in intestinal antioxidant capacity among pig breeds, and thus no comparisons could be made with other studies. Nevertheless, there have been some studies regarding meat antioxidant capacity among pig breeds, which exhibited differences of catalase and SOD in muscle among Pietrain, Landrace, Large-White, lberian, and lberian-Duroc [68]. Taken together, the digestion and absorption function in the three pig genotypes are distinct, which is accompanied by different digestive and absorptive enzymes activities, transport carriers abundance and antioxidant capacity in the intestine.

Pig breeds also differ for their intestinal barrier. Previous study has shown that Yorkshire pigs responded to LPS by increasing resistance (decreasing conductance), whereas resistance in Meishan intestine did not change with LPS, which indicated that intestinal barrier function in traditional pig breed was better than Yorkshire pigs [53]. Innate defenses, such as epithelial production of a-defensins and mucins, help prevent bacteria from crossing the mucosal barrier [69]. ZO-1 is a junctional adaptor protein that interacts with multiple other junctional components, including the transmembrane proteins of the claudin, occludin and JAM families, which is important for intestinal integrity and barrier function [70]. Furthermore, the direct host-commensal interaction is dictated by the presence of the intestinal mucus layer, and the expression and activation of mucin and ZO-1 are induced by exposing to a developing gut microbial community or their structural components, as well as the presence of products of bacterial metabolism [1]. In our study, the relative ZO-1 mRNA expression of the ileum in TP and TFM was higher than that in YP and YFM, and RP and RFM had the highest relative MUC2 mRNA expression in the duodenum, suggesting native pig breeds (TP and RP) had better gut barrier function than foreign pig breeds (YP), which verified their stronger adaptability and disease resistance. As mentioned above, the differences in intestine characteristics including intestinal development and gut barrier among pig breeds could transmit to their recipients by fecal microbiota transplantation, and the intestinal microorganisms are essential for intestine development of mammals.

Our study showed that the microbiota affected gut characteristics through their impacts on epithelial renewal rate, morphology, nutrient digestion and absorption, and the intestinal barrier. Thus, while bacteria in the gut are highly variable, the influence of the microbiota in the intestine has far-reaching effects on host physiology. Short chain fatty acids (SCFA) were generated by bacterial fermentation of dietary polysaccharides, and it significantly affects energy metabolism, intestine morphology and immune function of the host via activation of its receptor GPR41 and GPR43 [71, 72]. Moreover, gut microbiota play a key role in determining bile acid (BA) profiles and subsequent effects on host gene expression [73]. BA was reported to differentially activate BA receptors, which function as systemic signaling molecules to regulate host metabolism [74]. Gut microbiota affect pig characteristics and transfer these phenotypes to recipient mice via aforementioned mechanisms in the current study. This study provides new approaches to intervene in animal production and health.

## Conclusion

This study indicates that the microbiota composition differed among pig breeds, and TP had better intestinal morphology and barrier function than YP. The differences in gut-phenotypes among pig breeds would partially convey to recipient mice by fecal microbiota transplantation, especially intestinal morphology and enzyme activities. It is concluded that intestinal microbiota could transfer host gut characteristics from pigs to mice.

## Additional file

Additional file 1: Table S1. Primer sequences and annealing temperature. **Table S2.** Effect of genotype on phyla level in microbiota of pigs based on16S rRNA gene analysis. **Table S3.** Effect of genotype on genus level in microbiota of pigs based on16S rRNA gene analysis. **Table S4.** Effect of gut flora source on phyla level in microbiota of mice based on16S rRNA gene analysis. **Table S5.** Effect of gut flora source on genus level in microbiota of mice based on16S rRNA gene analysis. (DOCX 39 kb)
