# Erratum to: Intestinal microbiota could transfer host Gut characteristics from pigs to mice

**DOI:** 10.1186/s12866-016-0879-0

**Published:** 2016-10-31

**Authors:** H. Diao, H. L. Yan, Y. Xiao, B. Yu, J. Yu, J. He, P. Zheng, B. H. Zeng, H. Wei, X. B. Mao, D. W. Chen

**Affiliations:** 1 Institute of Animal Nutrition, Sichuan Agricultural University, Key Laboratory for Animal Disease-Resistance Nutrition of China Ministry of Education, Xinkang Road 46#, Ya’an, Sichuan Province 625014 People’s Republic of China; 2 Department of Laboratory Animal Science, College of Basic Medical Sciences Third Military Medical University, Gaotanyan Street, Chongqing, 400038 People’s Republic of China

## Erratum

Following the publication of this article [1] it was brought to our attention that incorrect funder’s number was included for the National Natural Science Foundation of China. The correct number is 3167131062 instead of the current 81370906.
