# Supplementary material for: Intestinal microbiota could transfer host Gut characteristics from pigs to mice
Source: BMC Microbiol. 2016 Oct 11;16:238. doi: 10.1186/s12866-016-0851-z (PMC5057279; doi:10.1186/s12866-016-0851-z)
Supplement: Additional file 1: Table S1. — Primer sequences and annealing temperature. Table S2. Effect of genotype on phyla level in microbiota of pigs based on16S rRNA gene analysis. Table S3. Effect of genotype on genus level in microbiota of pigs based on16S rRNA gene analysis. Table S4. Effect of gut flora source on phyla level in microbiota of mice based on16S rRNA gene analysis. Table S5. Effect of gut flora source on genus level in microbiota of mice based on16S rRNA gene analysis. (DOCX 39 kb) [file 12866_2016_851_MOESM1_ESM.docx]

Table S1 Primer sequences and annealing temperature

| Target gene | Forward primer 5’-3’ | Reverse primer 5’-3’ | Product length | Accession number |
| --- | --- | --- | --- | --- |
| Pigs |  |  |  |  |
| EGF | ATCTCAGGAATGGGAGTCAACC | TCACTGGAGGATGGAATACAGC | 165 | NM_214020.1 |
| GLP-2 | ACTCACAGGGCACGTTTACCA | AGGTCCCTTCAGCATGTCTCT | 149 | NM_005671883.1 |
| ANG4 | ACCACTTGTACGCACTCAGG | ACTCATCGAAGTGGACAGGC | 118 | NM_001163409.1 |
| IGF-1 | CTGAGGAGGCTGGAGATGTACT | CCTGAACTCCCTCTACTTGTGTTC | 137 | NM_001097417.1 |
| IGF-1R | TTCGCCAGATCCTAGGGGAG | TCCCAGCTTTGATGGTCAGG | 120 | NM_214172.1 |
| SGLT-1 | GCAACAGCAAAGAGGAGCGTAT | GCCACAAAACAGGTCATAGGTC | 137 | NM_001164021.1 |
| GLUT-2 | GACACGTTTTGGGTGTTCCG | GAGGCTAGCAGATGCCGTAG | 149 | NM_001097417.1 |
| SLC7A1 | TCTTTGCAGGTCGTTTGGGA | GGCTGATCACCTGTTGGAGT | 137 | [NM_001012613.1](http://www.ncbi.nlm.nih.gov/entrez/viewer.fcgi?db=nucleotide&id=60302865) |
| DMT1 | GCAGGTGGTTGACGTCTGTA | CACGCCCCCTTTGTAGATGT | 100 | NM_001128440.1 |
| ZnT1 | TGCTCTGCATGCTGTTACTGA | TGGAAGGAGTCCGAGAGCAT | 97 | NM_001139470.1 |
| CDX2 | GGAAACCTGGACCCACCAAA | TCCCTCACACCAAACACCAC | 114 | [NM_001278769.1](http://www.ncbi.nlm.nih.gov/entrez/viewer.fcgi?db=nucleotide&id=523580049) |
| Occludin | CAGGTGCACCCTCCAGATTG | GGACTTTCAAGAGGCCTGGAT | 110 | [NM_001163647.2](http://www.ncbi.nlm.nih.gov/entrez/viewer.fcgi?db=nucleotide&id=402746997) |
| ZO-1 | CTGAGGGAATTGGGCAGGAA | TCACCAAAGGACTCAGCAGG | 105 | [XM_013993251.1](http://www.ncbi.nlm.nih.gov/entrez/viewer.fcgi?db=nucleotide&id=927096856) |
| REG3γ | GGCTTGGAACCAAATGCTGG | TAGCCAGGGTATGAGCTGGT | 101 | XM_005662419.1 |
| MUC1 | GTGCCGCTGCCCACAACCTG | AGCCGGGTACCCCAGACCCA | 141 | [XM_001926883.5](http://www.ncbi.nlm.nih.gov/entrez/viewer.fcgi?db=nucleotide&id=927121447) |
| MUC2 | GGTCATGCTGGAGCTGGACAGT | TGCCTCCTCGGGGTCGTCAC | 181 | [XM_013989745.1](http://www.ncbi.nlm.nih.gov/entrez/viewer.fcgi?db=nucleotide&id=927100665) |
| β-actin | TCTGGCACCACACCTTCT | TGATCTGGGTCATCTTCTCAC | 114 | DQ178122 |
| Mice |  |  |  |  |
| EGF | CGTTGTTAGCACCATCCCTCA | GCACAACCAGGCAAAGGATG | 94 | NM_031197.2 |
| GLP-2 | AATCTTGCCACCAGGGACTT | GTGACTGGCACGAGATGTTG | 110 | NM_019810.4 |
| ANG4 | TGGCTCAGAATGAAAGGTACGA | CTTTGCAAGGCGAGGTTAGC | 123 | [NM_001301424.1](http://www.ncbi.nlm.nih.gov/entrez/viewer.fcgi?db=nucleotide&id=673921616) |
| IGF-1 | CTGGAGATGTACTGTGCCCC | CCTGCACTTCCTCTACTTGTGT | 128 | [NM_010113.4](http://www.ncbi.nlm.nih.gov/entrez/viewer.fcgi?db=nucleotide&id=894216176) |
| IGF-1R | GTACCGGCACAACTACTGCT | GGCCCTTTATCACCACCACA | 123 | [NM_001314010.1](http://www.ncbi.nlm.nih.gov/entrez/viewer.fcgi?db=nucleotide&id=930155589) |
| SGLT-1 | GATTGGGGAACCACCCATGT | AGCTTCAGTCCCTGCCTTTC | 115 | [XM_006540645.2](http://www.ncbi.nlm.nih.gov/entrez/viewer.fcgi?db=nucleotide&id=755520749) |
| GLUT-2 | ACCGGGATGATTGGCATGTT | GGACCTGGCCCAATCTCAAA | 150 | NM_008100.3 |
| SLC7A1 | GTTTCCCATGCCCCGAGTTA | AAGAGGAAGGCCATCACAGC | 144 | [NM_183417.3](http://www.ncbi.nlm.nih.gov/entrez/viewer.fcgi?db=nucleotide&id=161760628) |
| DMT1 | GGCGGAGCCGAATCCTATT | TGCTGTAGGCAGGGTTGATG | 145 | NM_001146161.1 |
| ZnT1 | GCCAAACAAACAGCCCGAAT | GCGACCAGACAAGGACTTCA | 143 | NM-009579.3 |
| CDX2 | AGGCTGAGCCATGAGGAGTA | TGTCTTCCCCTGAGGTCCAT | 134 | [NM_007673.3](http://www.ncbi.nlm.nih.gov/entrez/viewer.fcgi?db=nucleotide&id=145301600) |
| Occludin | CCTCCACCCCCATCTGACTA | TCTGGGTATGATCGCTTGCC | 92 | [NM_008756.2](http://www.ncbi.nlm.nih.gov/entrez/viewer.fcgi?db=nucleotide&id=31982140) |
| ZO-1 | CCCGGACTTTTGTCCCACTT | CCACCGTCCGCATAAACATC | 105 | [NM_009386.2](http://www.ncbi.nlm.nih.gov/entrez/viewer.fcgi?db=nucleotide&id=254675276) |
| REG3γ | CAGACAAGATGCTTCCCCGT | GCAACTTCACCTTGCACCTG | 94 | NM_011260.1 |
| MUC1 | TCTTTCCAACCCAGGACACC | ACTGCCATTACCTGCCGAAA | 128 | [NM_013605.2](http://www.ncbi.nlm.nih.gov/entrez/viewer.fcgi?db=nucleotide&id=530537203) |
| MUC2 | TGTGAGGAGGATGGCACCTA | GGGCACAATCTCGGTCTTCA | 150 | [NM_023566.3](http://www.ncbi.nlm.nih.gov/entrez/viewer.fcgi?db=nucleotide&id=410442542) |
| β-actin | GCAAGCAGGAGTACGATGAGT | GGTGTAAAACGCAGCTCAGTA | 86 | NM-007393.3 |

Table S2 Effect of genotype on phyla level in microbiota of pigs based on16S rRNA gene analysis

| Items | TP | YP | RP | SEM | *P*-value |
| --- | --- | --- | --- | --- | --- |
| *Proteobacteria* | 3.281 | 3.254 | 2.820 | 0.332 | 0.568 |
| *Actinobacteria* | 0.119 | 0.166 | 0.202 | 0.029 | 0.198 |
| *Bacteroidetes* | 36.309^b^ | 50.834^a^ | 34.130^b^ | 0.772 | <.001 |
| *Cyanobacteria* | 1.188 | 0.906 | 0.982 | 0.092 | 0.145 |
| *Elusimicrobia* | 0.190^a^ | 0.046^b^ | 0.030^b^ | 0.015 | <.001 |
| *Fibrobacteres* | 0.486^a^ | 0.132^b^ | 0.058^b^ | 0.070 | 0.006 |
| *Firmicutes* | 39.195^b^ | 35.340^c^ | 45.386^a^ | 0.560 | <.001 |
| *Fusobacteria* | 0.744 | 0.786 | 0.798 | 0.044 | 0.674 |
| *Lentisphaerae* | 0.052 | 0.070 | 0.084 | 0.018 | 0.502 |
| *Planctomycetes* | 0.147 | 0.168 | 0.164 | 0.051 | 0.953 |
| *Spirochaetes* | 15.251^a^ | 5.214^c^ | 11.802^b^ | 0.586 | <.001 |
| *Tenericutes* | 0.350^b^ | 0.826^a^ | 0.924^a^ | 0.090 | 0.004 |
| *Verrucomicrobia* | 1.331 | 1.268 | 1.428 | 0.173 | 0.810 |
| *WPS-2* | 0.005 | 0.126 | 0.058 | 0.048 | 0.262 |

TP, Tibetan pig; YP, Yorkshire pig; RP Rongchang pig.

^a-b^Within a row, means without a common superscript differ (*P* < 0.05).

Table S3 Effect of genotype on genus level in microbiota of pigs based on16S rRNA gene analysis

| Items | TP | YP | RP | SEM | *P*-value |
| --- | --- | --- | --- | --- | --- |
| *Prevotella* | 23.453^b^ | 34.579^a^ | 13.564^c^ | 1.618 | <.001 |
| *Succinivibrio* | 3.209^b^ | 6.889^a^ | 1.271^c^ | 0.190 | <.001 |
| *Treponema* | 16.862^a^ | 3.383^c^ | 11.372^b^ | 1.312 | 0.000 |
| *YRC22* | 0.596^c^ | 1.698^b^ | 9.496^a^ | 0.230 | <.001 |
| *Anaerovibrio* | 1.372^b^ | 2.236^a^ | 0.645^c^ | 0.167 | 0.001 |
| *Oscillospira* | 2.799 | 1.983 | 2.240 | 0.372 | 0.335 |
| *CF231* | 1.401^a^ | 0.953^b^ | 1.141^ab^ | 0.104 | 0.044 |
| *Megasphaera* | 0.105^b^ | 2.105^a^ | 0.035^b^ | 0.081 | <.001 |
| *Phascolarctobacterium* | 1.296 | 1.531 | 0.938 | 0.172 | 0.105 |
| *Mitsuokella* | 0.029^b^ | 0.801^a^ | 0.009^b^ | 0.042 | <.001 |
| *Lactobacillus* | 1.132^a^ | 0.498^b^ | 0.255^b^ | 0.139 | 0.006 |
| *Ruminococcus* | 0.481^b^ | 0.529^b^ | 1.562^a^ | 0.087 | <.001 |
| *02d06* | 0.484 | 0.475 | 0.921 | 0.126 | 0.060 |
| *Parabacteroides* | 1.250^a^ | 0.578^b^ | 0.545^b^ | 0.070 | 0.000 |
| *Paludibacter* | 0.326^b^ | 0.041^c^ | 0.501^a^ | 0.030 | <.001 |
| *Clostridium* | 0.280^a^ | 0.100^b^ | 0.140^b^ | 0.032 | 0.010 |
| *Bacteroides* | 0.984^a^ | 0.348^b^ | 0.820^a^ | 0.075 | 0.001 |
| *Roseburia* | 1.169^b^ | 0.232^c^ | 5.349^a^ | 0.113 | <.001 |
| *Dorea* | 0.143 | 0.206 | 0.187 | 0.021 | 0.152 |
| *Coprococcus* | 0.417 | 0.231 | 0.396 | 0.053 | 0.073 |
| *Sphaerochaeta* | 0.270 | 0.205 | 0.323 | 0.049 | 0.295 |
| *Blautia* | 0.869^a^ | 0.240^b^ | 0.539^b^ | 0.074 | 0.001 |
| *Acidaminococcus* | 0.026^b^ | 0.163^a^ | 0.007^b^ | 0.017 | 0.000 |
| *Fibrobacter* | 1.086^a^ | 0.172^b^ | 0.040^b^ | 0.087 | <.001 |
| *Campylobacter* | 0.044 | 0.053 | 0.024 | 0.010 | 0.191 |
| *SMB53* | 0.071^b^ | 0.122^a^ | 0.080^ab^ | 0.012 | 0.041 |
| *Akkermansia* | 0.288^a^ | 0.004^c^ | 0.115^b^ | 0.010 | <.001 |

TP, Tibetan pig; YP, Yorkshire pig; RP Rongchang pig.

^a-b^Within a row, means without a common superscript differ (*P* < 0.05).

Table S4 Effect of gut flora source on phyla level in microbiota of mice based on16S rRNA gene analysis

| Items | TFM | YFM | RFM | SEM | *P*-value |
| --- | --- | --- | --- | --- | --- |
| *Bacteroidetes* | 43.028^b^ | 52.871^a^ | 39.005^c^ | 0.450 | <.001 |
| *Firmicutes* | 48.386^b^ | 37.358^c^ | 52.357^a^ | 0.519 | <.001 |
| *Proteobacteria* | 3.083^a^ | 3.958^a^ | 1.240^b^ | 0.423 | 0.003 |
| *Actinobacteria* | 1.333^a^ | 0.770^b^ | 0.891^b^ | 0.067 | 0.001 |
| *Spirochaetes* | 1.759^a^ | 0.237^c^ | 1.486^b^ | 0.031 | <.001 |
| *Fusobacteria* | 1.227^b^ | 0.035^c^ | 1.984^a^ | 0.091 | <.001 |

TFM,Tibetan porcine flora-associated mice; YFM,Yorkshire porcine flora-associated mice; RFM,Rongchang porcine flora-associated mice.

^a-b^Within a row, means without a common superscript differ (*P* < 0.05).

Table S5 Effect of gut flora source on genus level in microbiota of mice based on16S rRNA gene analysis

| Items | TFM | YFM | RFM | SEM | *P*-value |
| --- | --- | --- | --- | --- | --- |
| *Acidaminococcus* | 2.238^a^ | 1.622^b^ | 0.018^c^ | 0.141 | <.001 |
| *Bacteroides* | 23.355^b^ | 33.974^a^ | 23.861^b^ | 1.091 | <.001 |
| *Bifidobacterium* | 1.766^a^ | 0.056^b^ | 0.071^b^ | 0.259 | 0.001 |
| *Blautia* | 13.243^a^ | 0.016^b^ | 1.255^b^ | 0.543 | <.001 |
| *Butyricicoccus* | 1.286^a^ | 0.006^b^ | 0.005^b^ | 0.079 | <.001 |
| *Butyricimonas* | 0.173^c^ | 1.134^a^ | 0.499^b^ | 0.061 | <.001 |
| *Clostridium* | 0.004^c^ | 0.540^b^ | 0.827^a^ | 0.024 | <.001 |
| *Coprococcus* | 2.380^a^ | 0.053^b^ | 0.050^b^ | 0.208 | <.001 |
| *Desulfovibrio* | 0.258^a^ | 0.004^b^ | 0.239^a^ | 0.016 | <.001 |
| *Eubacterium* | 0.741^a^ | 0.062^b^ | 0.039^b^ | 0.012 | <.001 |
| *Faecalibacterium* | 0.814^ab^ | 1.578^a^ | 0.042^b^ | 0.216 | 0.002 |
| *Fusobacterium* | 1.673^a^ | 0.000^b^ | 0.000^b^ | 0.167 | <.001 |
| *Lactobacillus* | 1.700^a^ | 1.355^b^ | 0.617^c^ | 0.064 | <.001 |
| *Mitsuokella* | 0.002^b^ | 0.000^b^ | 2.145^a^ | 0.043 | <.001 |
| *Oscillospira* | 0.267^b^ | 1.552^a^ | 0.043^b^ | 0.082 | <.001 |
| *Parabacteroides* | 1.318^a^ | 0.003^b^ | 0.003^b^ | 0.083 | <.001 |
| *Peptococcus* | 0.001^b^ | 0.532^a^ | 0.005^b^ | 0.027 | <.001 |
| *Phascolarctobacterium* | 0.0287^c^ | 1.454^a^ | 0.926^b^ | 0.058 | <.001 |
| *Prevotella* | 34.517^b^ | 41.557^a^ | 28.411^c^ | 0.700 | <.001 |
| *Roseburia* | 0.918^b^ | 0.645^b^ | 5.886^a^ | 0.379 | <.001 |
| *Ruminococcus* | 0.482^b^ | 0.092^c^ | 4.094^a^ | 0.068 | <.001 |
| *Staphylococcus* | 0.777^a^ | 0.058^b^ | 0.051^b^ | 0.071 | <.001 |
| *Streptococcus* | 0.441^a^ | 0.005^b^ | 0.002^b^ | 0.070 | 0.002 |
| *Sutterella* | 2.151^a^ | 0.728^b^ | 0.695^b^ | 0.140 | <.001 |
| *Veillonella* | 0.044^b^ | 0.000^b^ | 12.950^a^ | 0.376 | <.001 |

TFM,Tibetan porcine flora-associated mice; YFM,Yorkshire porcine flora-associated mice; RFM,Rongchang porcine flora-associated mice.

^a-b^Within a row, means without a common superscript differ (*P* < 0.05).
